# Mid-term results of hindfoot arthrodesis with a retrograde intra­medullary nail in 24 patients with diabetic Charcot neuroarthropathy

**DOI:** 10.1080/17453674.2020.1746605

**Published:** 2020-04-01

**Authors:** Mehmet Ersin, Mehmet Demirel, Mehmet Chodza, Fuat Bilgili, Onder Ismet Kiliçoglu

**Affiliations:** Department of Orthopaedics and Traumatology, İstanbul School of Medicine, İstanbul University, İstanbul, Turkey

## Abstract

Background and purpose — Hindfoot arthrodesis using retrograde intramedullary nailing assumes a critical role in limb salvage for patients with diabetic Charcot neuro­arthropathy (CN). However, this procedure is compelling and fraught with complications in diabetic patients. We report the mid-term clinical and radiological outcomes of retrograde intramedullary nailing for severe foot and ankle deformity in patients with diabetic CN.

Patients and methods — Hindfoot arthrodesis was performed using a retrograde intramedullary nail in 24 patients (15 females) with diabetic Charcot foot. The mean age of the patients was 62 years (33–82); the mean follow-up was 45 months (24–70). The primary outcomes were rates of fusion, limb salvage, and complications.

Results — The overall fusion rate was 23/24, and none of the patients needed amputation. The rate of superficial wound infection was 4/24, and no deep infection or osteomyelitis was observed postoperatively.

Interpretation — For selected cases of diabetic CN with severe foot and ankle deformity, hindfoot arthrodesis using a retrograde intramedullary nail seems to be a good technique in achieving fusion, limb salvage, and avoidance of complications.

Acute Charcot neuroarthropathy (CN) of the foot and ankle is a consequence of the combined neuropathic process including sensory, motor, and autonomic peripheral nerves, which is clinically characterized by obvious swelling, bone destruction, and final healing with severe bone deformity (Pinzur and Noonan 2005). Bone deformities in CN may cause several problems ranging from an ulcer to osteomyelitis and eventually amputation (Alvarez et al. 1994, Aktaş et al. 2016). Although any clinical condition that gives rise to sensory or autonomic neuropathy can result in this debilitating disorder, diabetes mellitus (DM) is the leading cause of CN (van der Ven et al. 2009).

Many authors have highlighted the salvage role of hindfoot arthrodesis in nonbraceable, severe ankle and hindfoot deformities in patients with CN (Alvarez et al. 1994, Bennett et al. 2005, Pinzur and Noonan 2005, Pelton and Hofer 2006). Various methods, comprising intramedullary nailing (Pinzur and Noonan 2005), crossed compression screws, blade plate (Alvarez et al. 1994), and external fixation (Russotti et al. 1988) have been employed for hindfoot arthrodesis. Nonetheless, retrograde intramedullary nailing has been a universal method of yielding stability and generating a plantigrade, stable foot for CN (Pinzur and Kelikian 1997, Pinzur and Noonan 2005). Conversely, the general consensus in the literature is that in patients with diabetic CN, hindfoot and ankle fusions are compelling and fraught with infectious and non-infectious complications due to their neuropathy, diabetes, poor bone quality, and the high mechanical loadings of internal fixation implants (Pinzur and Kelikian 1997, Perlman and Thordarson 1999, Mendicino et al. 2004, Chahal et al. 2006, ter Gunne and Cohen 2009, Wukich et al. 2011, Caravaggi et al. 2012, Myers et al. 2012).

We present the mid-term clinical and radiological outcomes of retrograde intramedullary nailing for severe foot and ankle deformity in patients with diabetic CN.

### Patients and methods

26 consecutive patients with diabetic CN who underwent unilateral hindfoot arthrodesis using a retrograde intramedullary nail between 2009 and 2015 by a single surgeon were reviewed retrospectively. After excluding 2 patients due to lack of medical records, the remaining 24 were included in the present study. 2 different retrograde intramedullary nails were used, including the expert HAN (Synthes AG, Bettlach, Switzerland) in 23 patients and the Trigen Hindfoot Fusion Nail (Smith & Nephew, Memphis, TN, USA) in 1 patient. After institutional review board approval was obtained, all the patients enrolled in the study were recalled for final follow-up clinical and radiological evaluations.

24 patients (mean age, 62 years [33–82]; 15 females) were analyzed retrospectively based on the institution medical records including radiology, operative notes, information on demographic characteristics, discharge reports, progress notes, and final follow-up with radiographical and physical examination. The mean follow-up was 45 months (24–70). The average weight was 90 kg (65–120), and BMI was 33 (24–42). According to BMI, 18 patients were obese, 4 of whom were morbidly obese (BMI > 40). The underlying pathology was type 2 DM in 20 patients and type 1 in 4. The average duration of DM at the time of the surgery was 19 years (10–32), and all patients had uncontrolled DM with a mean HbA1c of 91 mmol/mol (10.5%).

The preoperative comorbidities, as a well-established predictor of increased risk for surgical complications, were documented and graded according to the ASA system; all patients were grade III. The data regarding comorbidities consisted of history of smoking (n = 4), peripheral vascular disorder (n = 1), coronary artery disorder (n = 4), chronic renal failure (n = 2), morbid obesity (n = 4), and chronic obstructive pulmonary disease (n = 5). Furthermore, prior surgeries and previous forefoot amputation on the involved foot and ankle were documented as well.

The primary indications for hindfoot arthrodesis were bone deformity accompanied by nonhealing ulcers or severe instability. Our preoperative management protocol included an ulcer-free approach and thorough glycemic regulation. To obtain sufficient fixation, all patients were typically operated in the late stage of coalescence phase (stage II) or consolidation phase (stage III) according to the modified Eichenholtz classification (Figure 1) (Rosenbaum and DiPreta 2015). Multilayer compression bandaging was applied in patients with lymphedema until the operation. Additionally, 5 patients underwent an off-loading regimen with a non-weight-bearing circular cast due to infected ulcers before the operation. Of these, 2 patients were monitored in hospital with vacuum-assisted therapy, local debridement, and antibiotic administration. All the patients were ulcer-free at the time of the surgery.

**Figure 1.**
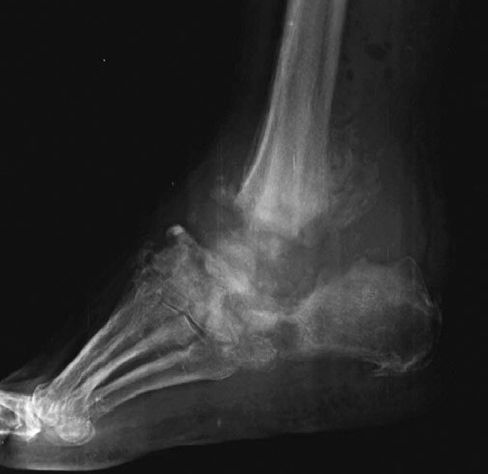
A case of severe CN with talar collapse.

The primary outcomes of the study were rates of fusion, limb salvage, and complication. Fusion was assessed radiographically and clinically in all cases. Solid fusion was identified as an osseous bridging across the arthrodesis site—at least 3 cortices in the serial radiographs. Recurrent deformity and an ability to bear weight on the affected foot with or without support were investigated in the physical examination. Intra- and postoperative complications were recorded.

Functional status and quality of life were additional secondary outcomes, which were measured using the AOFAS score including 40 points for pain, 50 points for function, and 10 points for alignment (Kitaoka et al. 1994), the Mazur Ankle Arthrodesis Score (Mazur et al. 1979), and the SF-36 Health Survey Questionnaire (Ware et al. 1998). The AOFAS scale was determined preoperatively and at the final follow-up examination; the Mazur ankle arthrodesis score and the SF-36 were conducted at the final follow-up.

Moreover, the ambulatory status of patients was evaluated on admission and at the final follow-up examination and divided into 3 categories: ambulation with a wheelchair (non-ambulation), ambulation with gait assistance devices (walker or crutches), and independent ambulation.

#### Surgical technique

The patients were positioned in either lateral decubitus or a supine position on a radiolucent operating table under general or spinal anesthesia. The C-arm was positioned to obtain imaging of subtalar and tibiotalar joints in both anteroposterior and lateral views. A pneumatic thigh tourniquet was routinely applied during the whole operation. Surgery started with a transfibular approach to the ankle. An osteotomy of the lateral malleolus, 2–4 cm proximal to the joint line, was performed using a sagittal saw with a second proximal shortening cut of at least 1 cm.

In the preparation of articular surfaces, the distal articular surface of the tibia was resected using an oscillating saw in lateral to medial direction. The superior articular surface of the talus (if present) was resected parallel to the distal tibial cut while holding the foot in plantigrade position. Then, all the cartilage, necrotic or sclerotic bone was removed from the subtalar or tibiotalar joint using curettes until viable subchondral bone was achieved. Furthermore, to enhance the fusion, multiple holes were drilled in the subchondral bone. In 9 patients with necrosis or collapse of the talus, remnants of the talar body were resected completely to achieve a stable tibiocalcaneal contact. In 3 patients, Chopart’s joint fusion was performed using cannulated screws in addition to hindfoot fusion.

After preparation of the surfaces, the ankle was positioned in neutral dorsiflexion–plantar flexion, and the foot was then realigned in neutral to 10° of valgus at the heel and mild posterior displacement of the calcaneus on the tibia in all cases. In some patients, the realignment was maintained temporarily with Kirschner wires.

In the later stage, the expert HAN in 23 patients and Trigen Hindfoot Fusion Nail in 1 patient were implanted according to their surgical technique guide. In tibiotalocalcaneal arthrodesis, the entry point was located at the intersection of lines along the center of the tibial canal in the lateral view and the lateral column of the calcaneus in the anteroposterior view. However, for tibiocalcaneal arthrodesis, the entry point was shifted slightly to a more posterior location in the calcaneus relative to the typical entry point.

In all patients who underwent the expert HAN, 1 spiral blade and 1 locking screw were first inserted from the posterior aspect into the calcaneus, followed by manual compression using a hammer. In the patient who underwent the Trigen Hindfoot Fusion Nail, 2 locking screws were used. Local autogenous bone graft was inserted in 3 cases. Allograft was not used. In 19 cases, the system was locked with 2 static locking screws from the lateral into the tibial diaphysis, and talar locking was not routinely used. At the final step of the operation, in order to enhance the stability, the lateral half of the fibula osteotomized in the initial stage was fixed with 2 cortical screws as a live biological plate. As an additional procedure, Achilles tendon lengthening was needed for only 1 patient with a severe contracture. Finally, anatomical foot alignment was confirmed clinically and with the image intensifier.

Postoperatively, a non-weight bearing below-knee circular cast was used for a minimum of 3 months. Then, full weight-bearing with a ROM walker was allowed in all patients for at least 6 months postoperatively. Healing enhancement methods were not used in this case series.

#### Ethics, funding, and potential conflicts of interest

Institutional review board approval was obtained (registration number 2019-0306). No funding for this study was received. Each author certifies that he or she has no commercial associations that might pose a conflict of interest.

## Results

**Figure 2.**
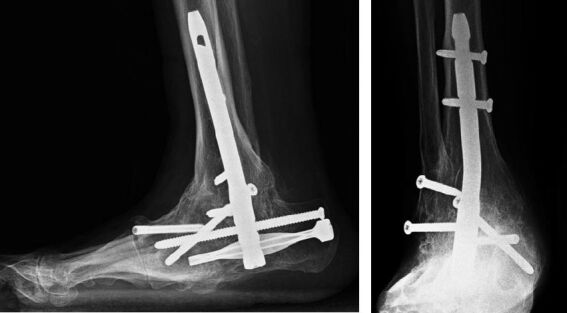
Solid fusion after retrograde intramedullary nailing using the expert HAN.

**Figure 3.**
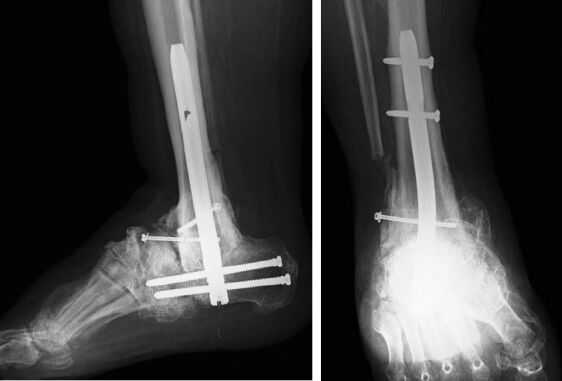
Solid tibiocalcaneal fusion using the Trigen Hindfoot Fusion Nail.

The demographic data and preoperative comorbidities of all the patients are summarized in the Table. Radiographic solid fusion was obtained with retrograde intramedullary nailing in 23 of 24 patients (Figure 2 and 3), with an average time to fusion of 10 months (6–14). In the remaining patient who underwent tibiotalocalcaneal arthrodesis there was no radiographic evidence of solid fusion at follow-up, but revision was not considered because the patient remained asymptomatic. All the patients were able to bear weight on the affected foot with or without support; physical examination revealed no recurrent deformity. No patient underwent amputation. During postoperative follow-up, four patients with superficial infection (4/24) were treated successfully with antibiotic and local debridement; none of the patients developed deep soft tissue infection or osteomyelitis. In addition, there was no need for revision surgery; however, the spiral blade in 1 patient and the calcaneal locking screw in 2 patients were removed due to retrocalcaneal bursitis.

In the evaluation of functional status, the mean AOFAS score increased from 40 (35–45) before surgery to 68 (42–86) after surgery. The mean Mazur ankle arthrodesis score was 64 (13–88) at the final follow-up. In terms of SF-36, the mean physical component score (PCS) was 70 (5–100), and the mean mental component score (MCS) was 76 (20–96) at the final evaluation.

Regarding the ambulatory status of patients, on admission 2 patients could ambulate independently without an assistance device; 11 patients required gait assistance devices (walker or crutches), and 11 patients were unable to walk. At the final follow-up visit, except for 1 patient who was non-ambulatory, all remaining patients could ambulate with a rocker-bottom shoe. Of these, half were able to walk independently and the other half with the aid of a gait device.

## Discussion

As an alternative approach to amputation, arthrodesis assumes a critical role in limb salvage for Charcot patients (Pinzur and Kelikian 1997, Pinzur and Noonan 2005, van der Ven et al. 2009). Although retrograde intramedullary nailing is a widely accepted method of hindfoot and ankle fusion to yield stability and generate a plantigrade and braceable foot (Pinzur and Kelikian 1997, Pinzur and Noonan 2005, Wukich et al. 2011), the general consensus in the literature is that this procedure is compelling but fraught with complications in diabetic patients (Mendicino et al. 2004, Caravaggi et al. 2006, Chahal et al. 2006, Wukich et al. 2011, Myers et al. 2012). Furthermore, according to our literature review, there has been little research (Pinzur and Kelikian 1997, Mendicino et al. 2004, Pinzur and Noonan 2005, Caravaggi et al. 2006, Pelton et al. 2006, Caravaggi et al. 2012, Chraim et al. 2018) to review the results of retrograde hindfoot arthrodesis nailing in diabetic patients with severe foot and ankle deformity. Therefore, the present study focused specifically on a certain group of patients with diabetic Charcot foot and reflected the results of intramedullary nailing for this challenging combination of disorders.

To provide additional evidence of feasibility and effectiveness of retrograde hindfoot arthrodesis in such patients, the primary outcomes of our study included fusion, limb salvage, and complication rates, concerning which the existing literature presents differing results. A study reported fusion in 19 patients and a fusion time of 20 months at a follow-up of 12–31 months in 20 diabetic patients with CN, despite 1 amputation (Pinzur and Kelikian 1997). Nevertheless, the authors stated that half of the patients, who needed talectomy to obtain acceptable tibiocalcaneal alignment, sustained several complications such as ulcers and wound infections. A recent study (Chraim et al. 2018) referred to a high fusion rate, 16 of 19 diabetic Charcot feet healed but with complications in 9 patients, and 3 patients progressed to amputation due to persisting infection and osteomyelitis.

In our opinion, glycemic regulation before surgery may be another important factor in preventing postoperative complications in our case series since it has been elucidated that increasing levels of Hgb A1c are closely associated with postoperative infections after hindfoot and/or ankle arthrodesis in diabetic population (Myers et al. 2012). Although we failed to attain the desired level of Hgb A1c in each patient postoperatively, all blood glucose levels returned to normal preoperatively in all patients with readjustment of anti-diabetic medication. Furthermore, considering patients’ high burden of comorbidities, substantive functional limitations according to ASA, and poor long-term glucose control, it may be difficult to obtain an adequate level of HbA1c.

We recommend utilizing second-generation IM nails as used in our study, which offer specific ankle- and foot-locking options as well as a compression mode, to provide sufficient stability and axial compression on arthrodesis sites, thus promoting fusion (Klos et al. 2009, Yakacki et al. 2011). Conversely, using such nail designs for hindfoot arthrodesis may need advanced technical skills.

Also, it is necessary to discuss the functional status and quality of life. The patients’ preoperative functional status and quality of life were obviously impaired, as reflected by the AOFAS and SF-36 PCS and MCS. The mean AOFAS score was improved from 40 (35–45) preoperatively to 68 (42–86) postoperatively; the mean Mazur ankle arthrodesis score was 64 (13–88) at the final follow-up. The SF-36 PCS and MCS were 70 and 76 respectively at the final examination. Considering the AOFAS improvement following arthrodesis, hindfoot arthrodesis provided a significant amelioration in patients’ functional status and satisfaction.

Some limitations should be mentioned when interpreting the findings of our study. The main limitations were the retrospective nature of the study and the relatively small number of patients. Nevertheless, the number of diabetic patients on whom we performed our analysis was consistent with most of the studies cited. Additionally, the follow-up period in this study was limited to the mid-term results. Ultimately, this study did not include a control group.

## Conclusion

For selected cases of diabetic CN with severe foot and ankle deformity, hindfoot arthrodesis using a retrograde intramedullary nail results in fusion, limb salvage, and a significant amelioration of quality of life. In addition, a thorough preoperative management protocol, including an ulcer-free approach and glycemic regulation, may minimize the rate of postoperative complications.

**Table:** Demographic data and preoperative comorbidities of the 24 patients

Median age at time of surgery	62 (33–82)
Male/female sex	9/15
Mean follow-up, months	45 (24–70)
Average weight	90 (65–120)
BMI	33 (24–42)
Average duration of DM, years	19 (10–32)
Mean HbA1c	10.5%
Smoking, n	4
Peripheral vascular disorder, n	1
Coronary artery disorder, n	4
Chronic renal failure, n	2
Morbid obesity, n	4
Chronic obstructive pulmonary disease, n	5

DM: diabetus mellitus; BMI: body mass index.
